# Evidence for Genotype-Associated Differences in Disease Severity and Limitations of Serotype-Based Classification in *Glaesserella parasuis* Revealed by Whole-Genome Sequencing in Japan

**DOI:** 10.3390/pathogens15060619

**Published:** 2026-06-09

**Authors:** Manao Ozawa, Motoshi Kawano, Shoko Iwamoto

**Affiliations:** National Veterinary Assay Laboratory, Ministry of Agriculture, Forestry and Fisheries, 2-1-22, Kannondai, Tsukuba 305-8535, Ibaraki, Japan; motoshi_kawano030@maff.go.jp (M.K.); shoko_iwamoto310@maff.go.jp (S.I.)

**Keywords:** antimicrobial resistance, *Glaesserella*, whole genome sequencing, vaccine

## Abstract

We conducted whole-genome sequencing to investigate serotypes, virulence-associated genes, antimicrobial resistance determinants, and genetic relationships among *Glaesserella parasuis* isolates from diseased pigs in Japan, focusing on underrecognized aspects of disease epidemiology and control. Although Glässer’s disease is well recognized in swine production, its epidemiology remains incompletely understood, particularly regarding the relationship between serotype, genotype, and pathogenicity. Serotypes 5 or 12 (5/12) (28.9%) were predominant, followed by serotype 7 (10.8%). Phylogenetic analysis based on core-genome single nucleotide polymorphisms and cluster analysis classified the isolates into three genetic groups, with no clear association between serotype and genetic grouping. One genetic group tended to exhibit a lower proportion of severe clinical cases compared with the others, with a statistically significant difference observed in one comparison but not in the other. These findings provide evidence suggesting genotype-associated differences in disease severity, indicating that pathogenic potential may be more closely linked to genetic background than to serotype. These findings suggest a potential limitation of serotype-based vaccine strategies. Although 86.7% of isolates lacked antimicrobial resistance genes, resistance determinants were identified on contigs predicted to be of plasmid origin. These results indicate that antimicrobial resistance, while not widespread, may be underestimated and could disseminate. Overall, our findings highlight underexplored aspects of Glässer’s disease relevant to improving control and prevention.

## 1. Introduction

*Glaesserella parasuis* causes Glässer’s disease, which results in pneumonia, fibrinous polyserositis, polyarthritis, and meningitis in pigs [1]. This opportunistic infection occurs due to factors such as transportation and sudden temperature changes [1]. Glässer’s disease is important because it causes economic losses in pig-raising areas globally [2].

Fifteen *G. parasuis* serotypes have been reported previously. Serotypes 4, 5, and 13 are prevalent globally [3,4,5,6]. Several untypable (UT) isolates have also been identified. Among *G. parasuis* isolates from sick pigs in Japan between 1987 and 1988, serotype 5 (14.2%) was the most prevalent, followed by serotype 4 (9.2%) [7]. It has been reported that serotypes 1, 5, 10, 12, 13, and 14 are highly virulent; serotypes 2, 4, 8, and 15 are moderately virulent; and serotypes 3, 6, 7, 9, and 11 are nonvirulent [8]. However, the relationship between serotype and pathogenicity remains inconsistent and poorly understood [9].

To date, several *G. parasuis* virulence factors have been identified. The relationship between the genotype and phenotype of *G. parasuis* has been analyzed using multiple-locus variable number tandem repeat analysis (MLVA), multilocus sequence typing (MLST), pulsed-field gel electrophoresis, and enterobacterial repetitive intergenic consensus polymerase chain reaction (ERIC-PCR), but no clear association has been found [5,10,11,12]. Furthermore, whole-genome sequencing (WGS) has recently been used to investigate virulence factors and other characteristics of clinical isolates [13,14,15,16]. Despite these advances, the relationship between genetic background and clinical outcome remains insufficiently explored.

Vaccination is generally the most effective way to control Glässer’s disease. Most commercial vaccines rely on whole-cell inactivated bacterins from one or two *G. parasuis* serotypes [3]. However, these vaccines frequently fail to provide sufficient cross-protection, because even when the infecting strain appears similar in serotype to the vaccine strain, small differences in the capsular polysaccharide can result in inadequate immunity [3,17]. Previous studies have demonstrated that serotype similarity does not necessarily confer protection against field strains, indicating an important but often overlooked limitation of serotype-based control strategies [3].

Antimicrobials are commonly used to treat Glässer’s disease, and treatment typically includes agents such as penicillins, macrolides, tetracyclines, phenicols, and fluoroquinolones [18]. However, the inappropriate use of antimicrobials leads to the selection of resistant bacteria on farms, as observed in *G. parasuis* isolates globally [19,20,21,22]. Nevertheless, antimicrobial resistance in *G. parasuis* has been relatively understudied in certain regions, including Japan.

Although serotypes and pathogenicity of *G. parasuis* isolates in Japan have been reported previously [7,23], comprehensive genomic analyses of field isolates remain limited. In particular, the relationships among genotype, pathogenicity, and antimicrobial resistance have not been systematically investigated using WGS. Therefore, this study aimed to elucidate the genetic characteristics of *G. parasuis* isolates from Japan, including virulence-associated genes and antimicrobial resistance determinants, using WGS, and to provide insights into underrecognized aspects of disease epidemiology and control.

## 2. Materials and Methods

### 2.1. Bacterial Isolates

We collected *G. parasuis* isolated and identified from pig-derived specimens that were sent to the Livestock Hygiene Service Centers nationwide for disease diagnosis. Eighty-three *G. parasuis* isolates were obtained from diseased pigs in 2013 (35 isolates), 2014 (17 isolates), 2015 (20 isolates), and 2022 (11 isolates) from 19 prefectures in Japan (Table S1). The 19 prefectures used for sample collection are located across the Hokkaido/Tohoku (HT), Kanto/Koshinetsu (KK), Tokai/Hokuriku (TH), Chugoku/Shikoku (CS), and Kyushu (KY) regions. All isolates were recovered from the lungs, heart, or brain of pigs with clinical signs of Glässer’s disease (death, respiratory symptoms, arthritis, neurological symptoms, and stunted growth). Each isolate was obtained from a single animal. Bacterial species were identified biochemically using the NN-20 Rapid ID test (SHIMADZU, Kyoto, Japan). *G. parasuis* isolates were classified according to the clinical information recorded during routine diagnostic investigations conducted by veterinarians at the Livestock Hygiene Service Centers. Classification was based on the predominant clinical outcome reported for each affected pig. Isolates were assigned to the death group (*n* = 34) when the animal died or was euthanized because of severe disease. The systemic group (*n* = 36) included pigs showing systemic manifestations compatible with Glässer’s disease, such as polyserositis, polyarthritis, meningitis, neurological signs, or severe generalized illness. The respiratory group (*n* = 13) included pigs presenting predominantly respiratory signs without reported systemic involvement. Clinical information was obtained from diagnostic records and interviews with farmers conducted by attending veterinarians. The isolation and identification of *G. parasuis* isolates were performed by the veterinarians of the Livestock Hygiene Service Centers in the prefectures.

### 2.2. Serotyping

The contig sequences of the isolates obtained using WGS were serotyped in silico using UniPro UGENE v45.1, an online PCR data analysis tool [24]. Previously reported primers were used [25].

### 2.3. Genome Sequencing and Analysis

Genomic DNA was prepared for WGS using a DNeasy Blood and Tissue Kit (Qiagen, Valencia, CA, USA). The 260/280 ratio of the extracted DNA was calculated using a Nanodrop instrument (Thermo Fisher Scientific, Waltham, MA, USA), and DNA samples with a ratio of 1.8 or higher were used. DNA samples were subjected to tagmentation and PCR amplification using a Nextera XT DNA Sample Preparation Kit (Illumina, Inc., San Diego, CA, USA). For library preparation, the DNA samples were diluted to 0.2 ng/μL. WGS was performed using a MiSeq instrument (Illumina, Inc., San Diego, CA, USA) in paired-end mode (2 × 300). De novo assembly was performed using Shovill software (version 1.0.4; https://github.com/tseemann/shovill, accessed on 21 August 2024). Contigs shorter than 300 bp were excluded from the Shovill assembly. To identify antimicrobial resistance genes, contig sequences were analyzed using ResFinder 4.0 (https://genepi.food.dtu.dk/resfinder, accessed on 21 August 2024). The identity and coverage thresholds used for ResFinder were 90% and 60%, respectively. *bcrA*, *bcrB*, *bcrC*, *bacA* (bacitracin resistance), and *bcr-1* (bicyclomycin resistance) were detected using the Comprehensive Antibiotic Resistance Database (https://card.mcmaster.ca, accessed on 21 August 2024) within ABRicate (version 1.0.1; https://github.com/tseemann/abricate, accessed on 2 September 2024). The identity and coverage thresholds used in ABRicate were 75% and 0%, respectively. Virulence-factor-encoding genes were identified using ABRicate software. A database including *bioB* (biotin synthase), F357_gp34 (Mu-like prophage protein gp36), *hicA* and *hicB* (corresponding to the toxin and antitoxin components, HicA and HicB, respectively), K756_02745 (bacteriophage protein/plasmid stabilization system protein), K756_06920 (CMP-N-acetylneuraminate-beta-galactosamide-alpha-2,3-sialyltransferase/lipopolysaccharide biosynthesis protein), and *capD* (polysaccharide biosynthesis protein) (GenBank accession No. KC795345.1) was constructed using previously reported sequences. These genes have been reported to be associated with *G. parasuis* isolated from pigs affected by Glässer’s disease [14,26]. Additionally, the Virulence Factor Database (http://www.mgc.ac.cn/VFs/, accessed on 2 September 2024) was used within ABRicate to detect genes encoding virulence factors [27]. The contigs (chromosomal or plasmid-derived) were classified using PlasFlow (version 1.1.0) [28]. The ratio of the core genome to the pan-genome was calculated using Prokka and Roary with default settings [29,30]. Species identity was further confirmed by average nucleotide identity (ANI) analysis using FastANI v1.34 (https://github.com/ParBLiSS/FastANI, accessed on 30 May 2024) against the genome of the *Glaesserella parasuis* type strain ATCC 19417^T^ (CCUG 3712; GCA_002015085.1).

### 2.4. Single-Nucleotide Polymorphism (SNP) Phylogenetic Analysis

Repeat and prophage regions of the reference genome (strain YHP1818, accession no. CP071487.1) were identified using NUCmer (MUMmer v3.23) [31] and PhiSpy v4.2.21 [32], respectively. Simulated short reads of contig sequences were generated using the Ngsngs program version 0.9.2 [33]. Simulated reads were aligned to the reference sequence using bwa-mem v0.7.17 [34] with default parameters. Mapping coverage and statistics were calculated using BEDTools v2.31.1 [35] and SAMtools v1.20 [36], respectively. Duplicate reads were marked using the MarkDuplicates tool from GAKT v4.5.0.0 [37], followed by variant calling with HaplotypeCaller. SNPs were extracted using GATK SelectVariants and filtered with VariantFiltration based on QD, QUAL, SOR, FS, MQ, MQRankSum, ReadPosRankSum, heterozygosity, and depth. Depth filtering applied a threshold of DP < max(mean depth/10, 5) to avoid excessive filtering in low-coverage regions. Base quality score recalibration was performed using GATK BaseRecalibrator and ApplyBQSR, followed by final variant calling and filtering. Recombination regions were predicted using consensus sequences constructed with BCFtools v1.21 [36] and BEDTools, followed by Gubbins v3.4 [38]. SNP datasets were merged using VCFtools v0.1.16 [39], and SNPs in non-core, repeat, recombination, and predicted prophage regions were excluded. Remaining SNPs were concatenated to construct a pseudo-sequence for core-genome phylogenetic analysis using IQ-TREE v2.4.0 [40] with parameters ‘-m MFP+ASC -b 100’. The phylogenetic tree was visualized using iTOL (version 6) [41].

### 2.5. Population Structure Analysis

Population structure was inferred using the Bayesian hierarchical clustering algorithm implemented in the R package fastbaps (version 1.0.7) (https://github.com/gtonkinhill/fastbaps, accessed on 28 January 2026). A core-genome alignment generated from single nucleotide polymorphisms (SNPs) created in 2.4 was used as input for the analysis. Clustering was performed in R (version 4.3.1) using fastbaps with default parameters [42]. The fastbaps function was applied to identify genetic population structure without prior assumptions regarding the number of clusters. Hierarchical Bayesian clustering was subsequently performed to resolve subpopulation structure at multiple levels. The resulting clusters were mapped to the phylogenetic tree for visualization and further interpretation of the population structure.

### 2.6. Statistical Analysis

Statistical analyses were performed using R software (version 4.3.1). Analysis of the association between the isolates and the clinical signs exhibited by the pigs from which they were isolated was evaluated using Fisher’s exact test. For post hoc pairwise comparisons, Fisher’s exact test was applied with Bonferroni correction for multiple testing. All tests were two-sided, with a significance level set at 0.05.

## 3. Results

### 3.1. Serotypes and Virulence Genes

Prior to downstream analyses, species identity was confirmed by average nucleotide identity (ANI) analysis against the genome of the *G. parasuis* type strain ATCC 19417^T^ (CCUG 3712). All isolates exhibited ANI values ranging from 96.2% to 98.9%, exceeding the accepted species threshold of 95–96%, thereby confirming their classification as *G. parasuis*. There were 27 isolates from Hokkaido/Tohoku, 32 from Kanto/Koshinetsu, 9 from Tokai/Hokuriku, 0 from Kinki, 2 from Chugoku/Shikoku, and 13 from Kyushu (Figure S1). Of the 83 isolates, 24 (28.9%) and 7 (10.8%) belonged to serotypes 5/12 (5 or 12) and 7, respectively. Other serotypes included 4 (9.6%), 1 (8.4%), and 13 (8.4%) (Table 1). Three isolates were identified and products were detected for both serotypes 1 and 2 using in silico PCR (serotype 1/2). UT isolates accounted for 25.3% of all isolates (Table 1).

The virulence genes and their respective prevalence among the 83 isolates were as follows: *gmhA* (83 isolates, 100%), *kdsA* (81 isolates, 97.6%), F33357_gp34 (71 isolates, 85.5%), *bioB* (62 isolates, 74.7%), K756_02745 (59 isolates, 71.1%), *capD* (55 isolates, 66.3%), group 1 *vtaA* (39 isolates, 47.0%), *hicA* and *hicB* (27 isolates, 32.5%), K756_06920 (25 isolates, 30.1%) group 2 *vtaA* (17 isolates, 20.5%), and group 3 *vtaA* (9 isolates, 10.8%) (Table 2).

### 3.2. Phylogenetic Analysis

Table S2 presents the assembly statistics for all 83 *G. parasuis* isolates analyzed in this study. The contig numbers ranged from 83 to 203, with N50 values between 31,421 and 97,951 bp, and mean coverage values generally exceeding 50× across isolates. The size of the pan-genome was 5821 genes in the 83 isolates used in this analysis. Of these, 1345 genes were found within all isolates. The percentage of core genes was 23.1% (1345 genes/5821 genes).

**Figure 1:**
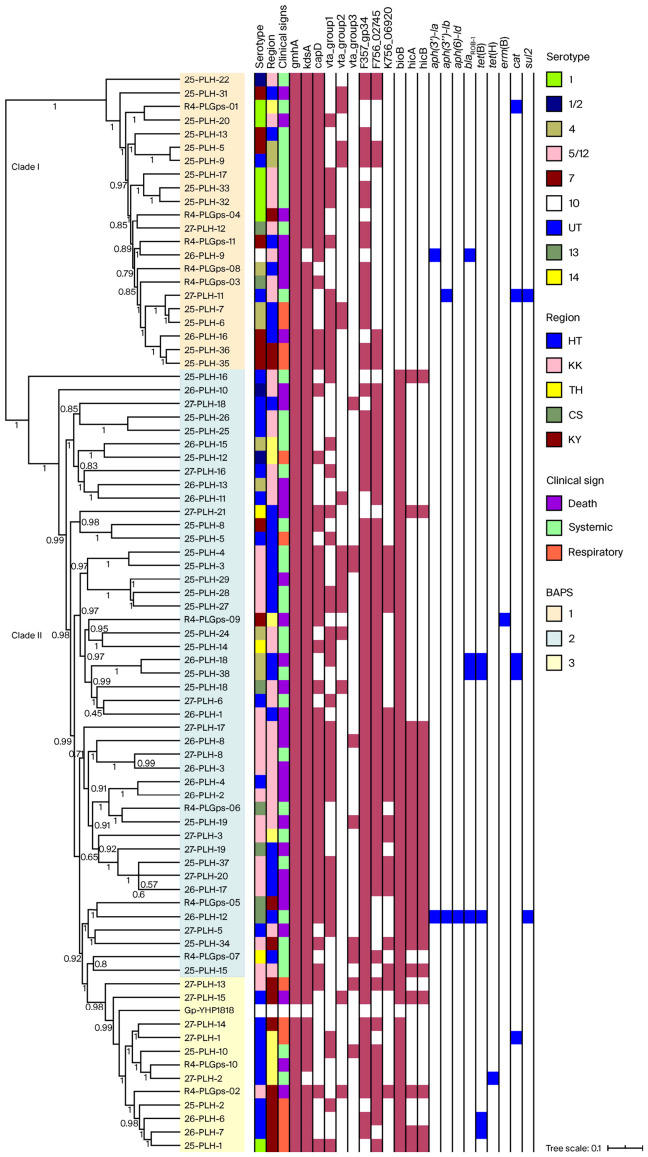
Genetic relatedness, epidemiology, virulence gene profile, and resistance gene profile of *Glaesserella parasuis* isolates (*n* = 83). Aminoglycoside-resistance genes: *aph(3′)-Ia*, *aph(3″)-Ib*, *aph(6)-Id*. Tetracycline-resistance genes: *tet*(B), *tet*(H). β-lactamase gene: *bla*_ROB-1_. Macrolide-resistance gene: *erm*(B). Chloramphenicol-resistance gene: *cat*. Sulfonamide-resistance gene: *sul2*. HT: Hokkaido/Tohoku district. KK: Kanto/Koshinetsu district. TH: Tokai/Hokuriku district. CS: Chugoku/Shikoku district. KY: Kyushu district. Reference: *G. parasuis* strain YHP1818 (GenBank accession no. CP071487.1). Bootstrap support values are represented at the branches. The red and blue blocks indicate the presence of virulence genes and resistance genes, respectively. The reference strain was not examined for serotype or gene carriage.

A phylogenetic tree of the 83 *G. parasuis* isolates derived from Japan was generated based on SNP sites, showing two well-supported clades (Figure 1). Clade 1 included isolates from all regions, whereas clade 2 included isolates from all regions except the Chugoku/Shikoku region. Genetically related isolates were identified in the same region of each clade.

Based on population structure analysis, all isolates were classified into one of three groups: 1, 2, and 3 (Figure 1). Group 1 belonged to clade 1 and did not have *bioB*, *hicA*, *hicB*, or K756_06920. The rates of the presence of group 1 *vtaA* genes were 50.0%, 49.0%, and 33.3% in groups 1, 2, and 3, respectively, and no significant differences were observed among the groups. Groups 1, 2, and 3 were dominated by serotype 7 isolates (31.8%, 7/22), serotype 5/12 isolates (44.9%, 22/49), and serotype UT isolates (75.0%, 9/12), respectively. Groups 1 and 2 contained a variety of serotypes. Group 3 consisted mostly of UT isolates, but also included isolates of serotype 1 and 5/12. Thus, there was no clear relationship between the groups and serotypes.

The relationship between the groups and clinical signs of the pigs from which each isolate was obtained was analyzed (Table 3). The rates of death, systemic symptoms, and respiratory symptoms were 36.4%, 45.5%, and 18.2% in group 1; 46.9%, 49.0%, and 4.1% in group 2; and 25.0%, 16.7%, and 58.3% in group 3, respectively. The combined rate of death and systemic symptoms was significantly lower in group 3 (41.7%) than in group 2 (95.9%). The relationship between serotype and clinical symptoms was such that the combined rates of death and systemic symptoms ranged from 66.7% to 100%; however, there was no significant difference in the rates between serotypes (Table S3).

### 3.3. Antimicrobial Resistance Genes

No resistance genes were detected in 72 of the 83 isolates (86.7%). The *tet*(B) and *cat* genes were dominant (6.0%, 5/83), followed by *bla*_ROB-1_ (4.8%, 4/83; Figure 1). Only one isolate (1.2%, 1/83) harbored the macrolide resistance gene, *erm*(B). A combination of aminoglycoside-, β-lactam-, tetracycline-, and sulfonamide-resistance genes was observed in one isolate.

The classification of contigs with resistance genes revealed six plasmid-derived contigs (Table S4), which harbored resistance genes for aminoglycosides, β-lactams, macrolides, phenicols, or sulfonamides. *aph(3′)-Ia*, *aph(3″)-Ib*, *aph(6)-Id*, and *sul2* were seen on the same plasmid-derived contig.

## 4. Discussion

*G. parasuis* is a major health threat to pig production and requires antimicrobial agent administration to control. Controlling *G. parasuis* involves multifaceted measures including vaccination, antimicrobials, and strict husbandry hygiene management. Information on the serotypes, virulence profiles, and predicted antimicrobial resistance patterns of *G. parasuis* obtained using WGS is useful for vaccine prevention and antimicrobial therapy. Furthermore, molecular epidemiological analysis using WGS helps estimate the transmission routes of *G. parasuis* in pig farms [15,16]. However, despite its importance, key aspects of Glässer’s disease, including the relationship between genotype and disease severity, limitations of serotype-based control, and the dynamics of antimicrobial resistance, remain insufficiently addressed.

In this study, serotypes 5/12 were the most common among *G. parasuis* isolates (Table 1). Serotype distribution varies depending on the country and region. Serotypes 4, 5, and 13 are common in China, Brazil, and Germany [21,22,43], whereas serotypes 7, 13, and 2 are found in North America [15]. Serotypes 4, 5/12, and 1 were previously reported in Europe [44]. Recently, serotype 7 isolates have been frequently detected in clinical cases in North America, Europe, and China [13,15,44]. Japan imports breeding pigs from North America and Europe [45]. Although the genotype of serotype 7 isolates in this study was not compared with the genotypes of isolates originating from North America, the possibility that serotype 7 isolates entered Japan through pig imports cannot be ruled out.

In the present study, we combined core-genome SNP analysis with Bayesian analysis of population structure (BAPS) to more precisely understand the population structure of *G. parasuis* [46]. While core-genome SNP-based phylogenetic analysis can capture vertical evolutionary history with high resolution, the extremely high recombination rate exhibited by *G. parasuis* [14] makes it difficult to accurately distinguish between actual phylogenetic groups and genetic population structures. Therefore, combining core-genome SNP analysis with BAPS, which statistically absorbs the effects of recombination and estimates genetically consistent clusters, may overcome the limitations of core-genome SNP analysis. In particular, for bacteria with high recombination rates, such as *G. parasuis*, phylogenetic proximity does not necessarily imply the same evolutionary group. Howell et al. [14] demonstrated that BAPS analysis divided a population into five clades, which corresponded to the two major clades in the phylogenetic tree. Gong et al. [13] also classified 764 strains into 10 BAPS groups that showed high concordance with clades 1/2 based on core-genome SNPs. In this study, clade 1 corresponded to BAPS group 1 and clade 2 corresponded to other BAPS groups. The concordance between the phylogenetic and BAPS clustering results supports the robustness of the inferred population structure despite the high recombination rate of G. *parasuis*.

In *G. parasuis*, the relationship between the presence of virulence factors and the manifestation of virulence remains unclear [14,47]. In this study, all isolates were classified into three groups based on phylogenetic and cluster analyses. Furthermore, a significant association between genetic grouping and clinical signs was observed in one comparison. Specifically, significantly fewer deaths and systemic symptoms were observed in group 3 than in group 2. These findings suggest that disease severity may be associated with genetic background., although statistical significance was observed in only one comparison, suggesting that the relationship between genotype and clinical outcome is complex and may be influenced by additional factors. Group 1 *vtaA* genes have been reported to be associated with the virulence of *G. parasuis* [48,49]. In this study, the prevalence of the group 1 *vtaA* genes was 33.3%, 45%, and 50% in BAPS groups 1, 2, and 3, respectively. Although the prevalence was lower in group 1 than in the other groups, this difference was not statistically significant. These results suggest that virulence in *G. parasuis* cannot be explained by *vtaA* alone and may instead be determined by a combination of multiple genetic factors, highlighting an underexplored aspect of genotype–phenotype relationships in this pathogen.

Several limitations should be considered when interpreting the observed association between genetic background and clinical severity. First, clinical classification was based on information collected during routine diagnostic investigations and was not derived from a standardized clinical scoring system. Therefore, some degree of subjectivity and inter-observer variability may have been present. Second, host-related factors that could influence disease outcome, including genetic background, breed susceptibility, management conditions, and immune status, were not available for analysis. Although information regarding vaccination history was available for some animals, these data were incomplete and not sufficiently standardized across all cases to permit meaningful statistical evaluation and were therefore not incorporated into the statistical analyses. Third, information regarding concurrent infections with immunomodulatory pathogens, such as porcine reproductive and respiratory syndrome virus (PRRSV) or porcine circovirus type 2 (PCV2), was unavailable. Such co-infections may influence disease severity independently of bacterial genotype. Consequently, the associations observed in this study should be interpreted as evidence of a potential relationship between genetic background and clinical outcome rather than definitive evidence of a causal relationship between genotype and disease severity.

Inactivated vaccines are used globally to prevent Glässer’s disease. However, the efficacy of these vaccines is limited by incomplete cross-protection between serotypes [2,17,50]. In this study, although no clear association was observed between serotype and genotype, clinical outcomes and the presence of virulence factors tended to be associated with genetic background. Furthermore, although not confirmed in this study, a correlation between the presence of virulence factors and genotype has been previously reported [15,51]. The mechanisms underlying cross-protection between serotypes remain poorly understood. Furthermore, many UT isolates may possess different types of polysaccharides or lack capsules. These findings suggest that serotype alone may not fully explain vaccine efficacy, and that genetic background, including virulence-associated genes, may influence protection. These observations highlight a limitation of serotype-based vaccine strategies and suggest that incorporating genetically diverse strains may improve protective coverage against clinical isolates, including UT strains. However, further experimental studies are required to verify this hypothesis.

In Japan, inactivated bacterins containing serotypes 2 and 5 are commercially available; however, their protective capability against all isolates within these serotypes remains uncertain, and cross-protection against heterologous serotypes appears limited [50]. These findings further emphasize the limitations of current serotype-based vaccination strategies and the need for careful consideration when applying these vaccines in the field. In addition to vaccination, strict hygiene and management practices are essential for effective prevention of Glässer’s disease.

Overall, 86.5% of the isolates did not harbor any resistance genes. However, reports from the United States and China have shown that almost all isolates possess *ksgA*, *bcr*, *bacA*, *sul2*, and *aph(3″)-Ib*, which confer resistance to kasugamycin, bicyclomycin, bacitracin, sulfonamide, and streptomycin, respectively [15,16]. However, Gong et al. [13] reported that the detection rates of *sul2* and *aph(3″)-Ib* were 12.7% and 9.82%, respectively. Mugabi et al. [15] used SRST2 [52] to detect resistance genes. In contrast, Gong et al. [13] used ResFinder. In the present study, we used ResFinder, and the detection rate of resistance genes was similar to that reported by Gong et al. Differences in resistance gene detection rates are thought to be largely influenced by differences in analysis tools, sequencing data quality, and bioinformatics analysis conditions. In fact, it has been pointed out that in antimicrobial resistance analysis using next-generation sequencing, data quality, the database used, and the choice of analysis tools have a significant impact on the reproducibility and accuracy of results [53]. However, previous studies and this study have not confirmed the phenotypes of isolates harboring resistance genes. Therefore, future studies should investigate the extent to which resistance genotypes and phenotypes match.

*G. parasuis* possesses *erm*(A), *erm*(B), and *erm*(T) genes, which confer macrolide resistance [13,15,54]. However, the detection rates of these genes among *G. parasuis* isolates are very low. Only one *G. parasuis* isolate obtained in 2022 possessed *erm*(B). The contig carrying this *erm*(B) gene was presumably of plasmid origin. This sequence was found to be homologous to 4 kbp of *G. parasuis*, *A. pleuropneumoniae*, and *Mannheimia haemolytica*-derived plasmids (coverage, 67%; identity, 99%) based on the Basic Local Alignment Search Tool (BLAST) (https://blast.ncbi.nlm.nih.gov/Blast.cgi, accessed on 5 September 2024) results. The remaining 33% were found to be homologous to plasmids possessing *erm*(B) from Gram-positive bacteria, such as *Enterococcus*, *Streptococcus*, and *Staphylococcus* (coverage, 27%; identity, 98%). Therefore, these sequences may have been inserted into the plasmids found within *G. parasuis*. 

Plasmids play an important role in the horizontal transfer of resistance genes. In this study, six contigs presumably derived from plasmids were detected, containing resistance genes, including those for β-lactams and aminoglycosides. Small plasmids of 5–7 kbp have previously been reported in *G. parasuis* [16,55], and some of the contigs in this study were of a similar size. Although we did not obtain the complete sequences of the plasmids in this study, they may be similar to previously reported small plasmids. To prevent the spread of such plasmids in *G. parasuis*, careful use of antimicrobials is required when treating Glässer’s disease.

In conclusion, *G. parasuis* isolates were divided into three groups based on phylogenetic and clustering analysis. Although we did not find a clear correlation between serotype and genotype, we observed a potential association between clinical symptoms and the genotype of some isolates. Our findings suggest genotype-associated differences in clinical severity and reinforce the limitations of serotype-based approaches to disease control. These findings suggest that vaccine efficacy cannot be reliably predicted based on serotype alone, highlighting an important limitation of current control strategies. In addition to vaccination, strict hygiene and management practices are essential for the effective prevention of Glässer’s disease. Although antimicrobial resistance genes may spread via plasmids or ICEs, only a few isolates possess resistance genes, indicating that antimicrobial resistance is not widespread among *G. parasuis* isolates in Japan. However, antimicrobial agents should be used carefully after confirming the susceptibility of the causative bacteria to prevent further spread of antimicrobial resistance. Overall, this study highlights that key aspects of Glässer’s disease—including genotype–pathogenicity relationships, vaccine limitations, and antimicrobial resistance dynamics—remain insufficiently understood and warrant further investigation.

## Figures and Tables

**Table 1:** Serotypes of *G. parasuis* (*n* = 83).

Serotype	Number of Isolates in				Total	%
	2013	2014	2015	2022		
1	5			2	7	8.4
1/2	2	1			3	3.6
4	4	3		1	8	9.6
5/12	10	5	8	1	24	28.9
7	5	2		2	9	10.8
10		1			1	1.2
13	1	1	2	3	7	8.4
14	1		1	1	3	3.6
UT	7	4	9	1	21	25.3
Total	35	17	20	11	83	100

**Table 2:** Serotypes and virulence genes of G. parasuis (*n* = 83).

Serotype	Virulence Genes												*n*
	*gmhA*	*kdsA*	*capD*	*vta*_group1	*vta*_group2	*vta*_group3	F357_gp34	K756_02745	K756_06920	*bioB*	*hicA*	*hicB*	
1	7	7	7	5	1		2	1		1	1	1	7
1/2	3	3	3				3	3		2			3
4	8	8		5	3		8	5		5			8
5/12	24	24	24	11	7	6	24	22	24	24	16	16	24
7	9	9	9	4	2		8	7		2			9
10	1		1										1
13	7	7	7	2	1		7	3		5	4	4	7
14	3	3	2	2		1	2	2		3	1	1	3
UT	21	20	2	10	3	2	17	15	1	19	5	5	21
Total	83	81	55	39	17	9	71	58	25	61	27	27	83

**Table 3:** Relationship between groups and clinical signs.

BAPS (Group)	Clinical Signs				Total
	Death (%)	Systemic (%)	Death + Systemic (%) *	Respiratory (%)	
1	8 (36.4)	10 (45.5)	18 (81.8) ^ab^	4 (18.2)	22
2	23 (46.9)	24 (49.0)	47 (95.9) ^a^	2 (4.1)	49
3	3 (25.0)	2 (16.7)	5 (41.7) ^b^	7 (58.3)	12

* Different letters indicate significant difference (*p* < 0.05).

## Data Availability

All raw whole-genome sequencing reads generated in this study have been deposited in the DNA Data Bank of Japan (DDBJ) Sequence Read Archive under BioProject accession number PRJDB19113.

## Supplementary Materials

The following supporting information can be downloaded at: https://www.mdpi.com/article/10.3390/pathogens15060619/s1, Figure S1: Geographical distribution of isolates included in this study across Japan; Table S1: *G. parasuis* isolates used in this study (*n* = 83); Table S2: Assembly statistics of G. *parasuis* (*n* = 83); Table S3: Relationship between serotypes and clinical signs; Table S4: Classification of contigs and resistance genes harbored in those contigs.
