# Supplementary figures and images for: Evidence for Genotype-Associated Differences in Disease Severity and Limitations of Serotype-Based Classification in Glaesserella parasuis Revealed by Whole-Genome Sequencing in Japan
Source: Pathogens. 2026 Jun 9;15(6):619. doi: 10.3390/pathogens15060619 (PMC13306171; doi:10.3390/pathogens15060619)

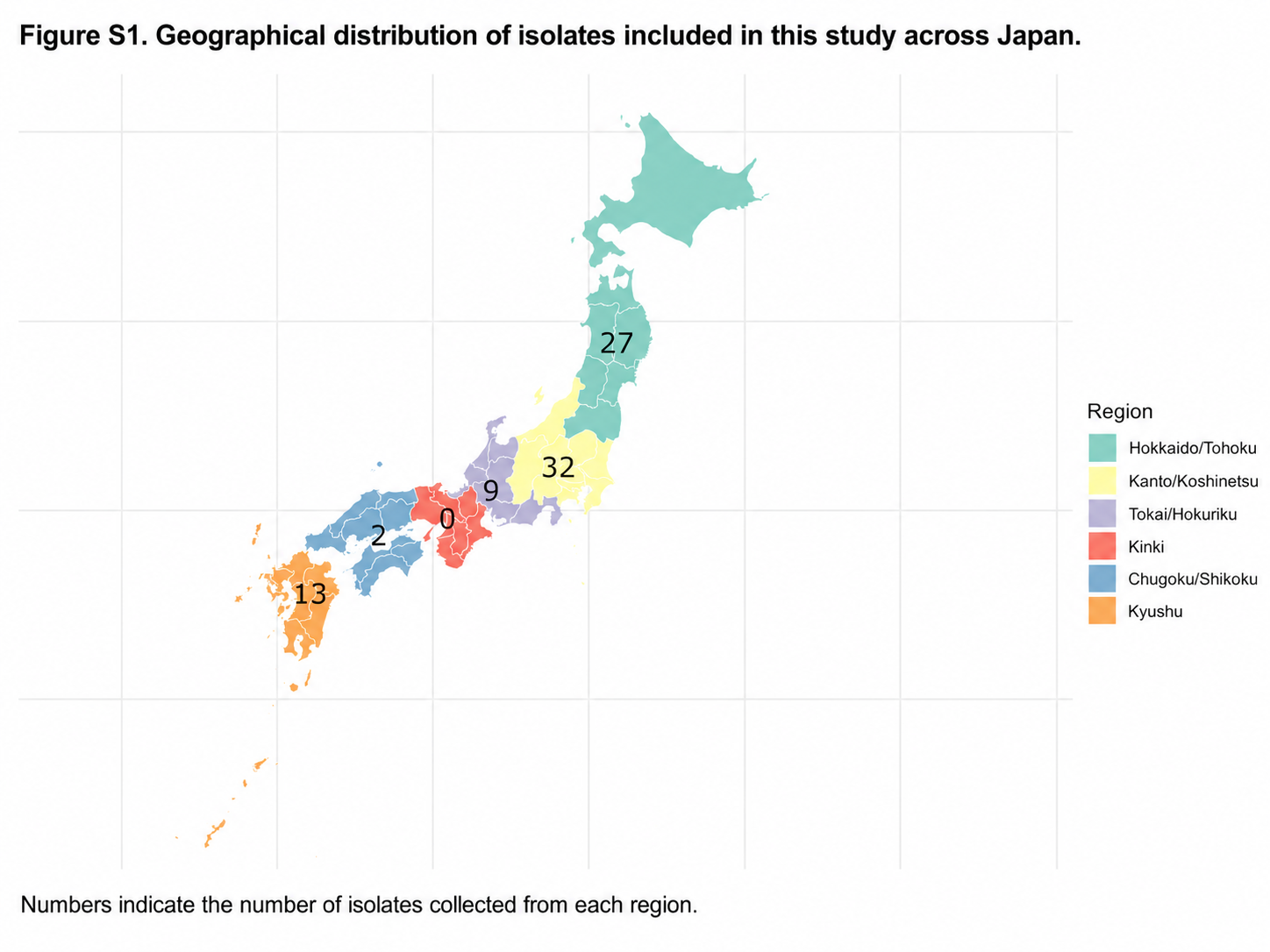

Supplement: Supplementary file 1 [file pathogens-15-00619-s001.zip › Figure_S1.png]
